# Ordered Avalanches on the Bethe Lattice

**DOI:** 10.3390/e21100968

**Published:** 2019-10-03

**Authors:** Malgorzata J. Krawczyk, Paweł Oświęcimka, Krzysztof Kułakowski, Stanisław Drożdż

**Affiliations:** 1 Faculty of Physics and Applied Computer Science, AGH University of Science and Technology, al. Mickiewicza 30, 30-059 Kraków, Poland; gos@agh.edu.pl; 2 Complex Systems Theory Department, Institute of Nuclear Physics, Polish Academy of Sciences, ul. Radzikowskiego 152, 31-342 Kraków, Poland; pawel.oswiecimka@ifj.edu.pl; 3 Faculty of Physics, Mathematics and Computer Science, Cracow University of Technology, ul. Warszawska 24, 31-155 Kraków, Poland; Stanislaw.Drozdz@ifj.edu.pl

**Keywords:** self-organized criticality, multifractals, mean field, social processes

## Abstract

We discuss deterministic sequences of avalanches on a directed Bethe lattice. The approach is motivated by the phenomenon of self-organized criticality. Grains are added only at one node of the network. When the number of grains at any node exceeds a threshold *b*, each of *k* out-neighbors gets one grain. The probability of an avalanche of size *s* is proportional to $s^{-\tau }$. When the avalanche mass is conserved (k=b), we get τ=1. For an application of the model to social phenomena, the conservation condition can be released. Then, the exponent τ is found to depend on the model parameters; τ ≈ log(b)/log(k). The distribution of the time duration of avalanches is exponential. Multifractal analysis of the avalanche sequences reveals their strongly non-uniform fractal organization. Maximal value of the singularity strength $\alpha_{max}$ in the bifractal spectrum is found to be 1/τ.

## 1. Introduction

Scientific interest in the phenomenon of self-organized criticality (SOC) is continuously growing since its initialization by the famous paper by Bak, Tang and Wiesenfeld in 1987 [1]. The phenomenon is attractive for its apparent ubiquity in different areas of knowledge, from physics of superconductors to earthquakes [2,3]. In addition, the theory of SOC promises classes of universality, analogously to theory of equilibrium phase transitions [4]. Despite this, even the definition of SOC remains debatable [2,3]. Basic ingredients of SOC have been summarized by Jensen as “slowly driven, interaction-dominated threshold systems” [2]. Under these watchwords, a rich set of concepts is hidden; for a detailed handbook, we refer to [3]. We believe that, in the face of an ambiguity of the main idea, simple analytical constructions are worth attention.

Our first aim here is to present such a construction, where main features of SOC are built-in in the form of assumptions. Besides that, the formulation is simplified as much as possible. In effect, the mathematical formalism remains at a simple level; the construction can be easily used as a toy model. Our second aim is to explore the model properties when it is applied to social phenomena, where the conservation of mass of avalanches—obvious for a sand pile—ceases its validity [5].

The idea of SOC is well established in the area of online social dynamics, in particular in the behaviour of bloggers [6,7] and in systems of collective knowledge [8,9]. In all these works, data have been gathered online and modeled with weighted bipartite networks of users and posts. In [6], a number of parameters has been introduced to describe avalanche dynamics of series of posts: distribution of time delays to posted material, fraction of negative comments, sum of weights of links to a given posts, and contingency of bloggers’ activity; these parameters have been collected from real data (apidoc.digg.com). In [7], the search has been broadened to networks of Ubuntu chats; the model analyses have been compared with empirical data. In particular, the Hurst exponents of the avalanche sequences have been found to be larger than 1/2 in all cases, which indicated a persistent character of the time series. Similarly, SOC behaviour has been identified in online data of a series of questions and answers, collected from math.stackexchange.com [8,9]. The exponent of the scale-free distribution of the avalanche size has been found to depend on details (as the users’ expertise), but it varied around the mean field value 3/2.

**Figure 1:**
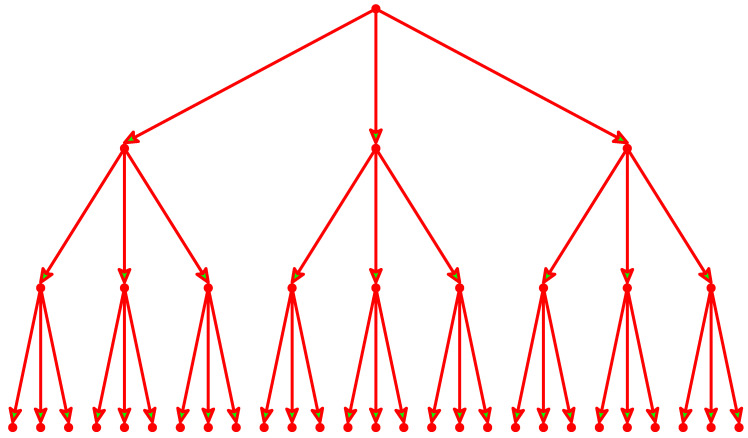
The directed Bethe lattice for k=3. The site on the top is the root, where grains are introduced to the system.

This study is less advanced with respect to contact with real data, and its advantage is its analytical character. We consider a directed and unweighted Bethe lattice, as in Figure 1, with nodes linked directly by arrows from the root represent messages posted as comments to an initial post. Each signal is initialized at the root. Subsequent posts appear as a response to the former comments and they form a next shell of nodes, and so on. The lattice is regular, which means that each post triggers the same number of reactions. As in sandpile models [3], the state of nodes is represented by a number of grains. Once this number at a given node exceeds some critical value, a toppling appears and grains are distributed down along the arrows. Breaking of conservation of grains means that the number of grains released by toppling is not necessarily equal to the number of nodes adopting grains; however, each neighbor gets one grain. Similarly, in a model social system, the number *k* of users who answer a given post is not necessarily equal to the number *b* of posts read by the user before he decides to write his own post. These two numbers *k* and *b* are the parameters of our model.

Our result is that, when the conservation does not hold, the exponent τ, defined via the avalanche mass distribution $P\left(s\right)\propto s^{-\tau }$ [3], is a function of the parameters. Such a dependence, rarely reported in the literature of SOC, is usually related to some amount of disorder in the sandpile model [10,11]. As disorder is absent in the approach presented here, it seems that our result is new. Furthermore, the fractal analysis reveals the scaling of the fluctuation functions $F_{q}\left(s\right)$, which allows for discussing properties of multifractal spectra.

In the next section, we formulate the model and list its analytical results. Section 3 is devoted to a generalization, motivated by an application to social phenomena, namely to the spreading of rumors. In this area of research, the conservation of mass of avalanches makes no sense. In Section 4, fractal properties of the signal are described. The last section is devoted to discussion, with some notes on possible applications of the model.

## 2. The Model and the Results

Consider a branch of a directed Bethe lattice, as shown in Figure 1. From each site, *k* directed links lead to *k* neighbors at the next level. Through the root at the top, a kind of units are supplied; their nature may remain unspecified, we note only that they are equivalent to grains of sand in the classical formulation of SOC [1]. For convenience, they will be termed grains from now on. The grains are collected at the root (level 1) until their number attains a threshold value *b*. Then, they are moved to the neighboring sites at level 2, one grain per each neighbor. This is an avalanche of size 1. In this section, we assume that the barrier *b* is equal to the number *k* of links outgoing from each site; in other words, the number of grains is conserved. Later, this assumption will be released. When the numbers of grains at the 2nd level attain *b*, all grains there are moved again, equally distributed among grains at the 3rd level. This is an avalanche of size 1+k. The next largest avalanche is $1+k+k^{2}$, and so on.

Here, we adopt the convention that the grain which initiates an avalanche is counted as a part of this avalanche. Then, in the simplest case of b=k=2, the sequence of sizes of avalanches is: 1,3,1,7,1,3,1,15,1,3,1,7,1,3,1,31,⋯. This sequence, known as A038712 [12], can be reproduced according to the following rule (taking k=2):

1. start with 1,
2. repeat the sequence k−1 times, then add $k^{p}$ to the last element, where *p* is the number of repetition,
3. go to 2.

**Figure 2:**
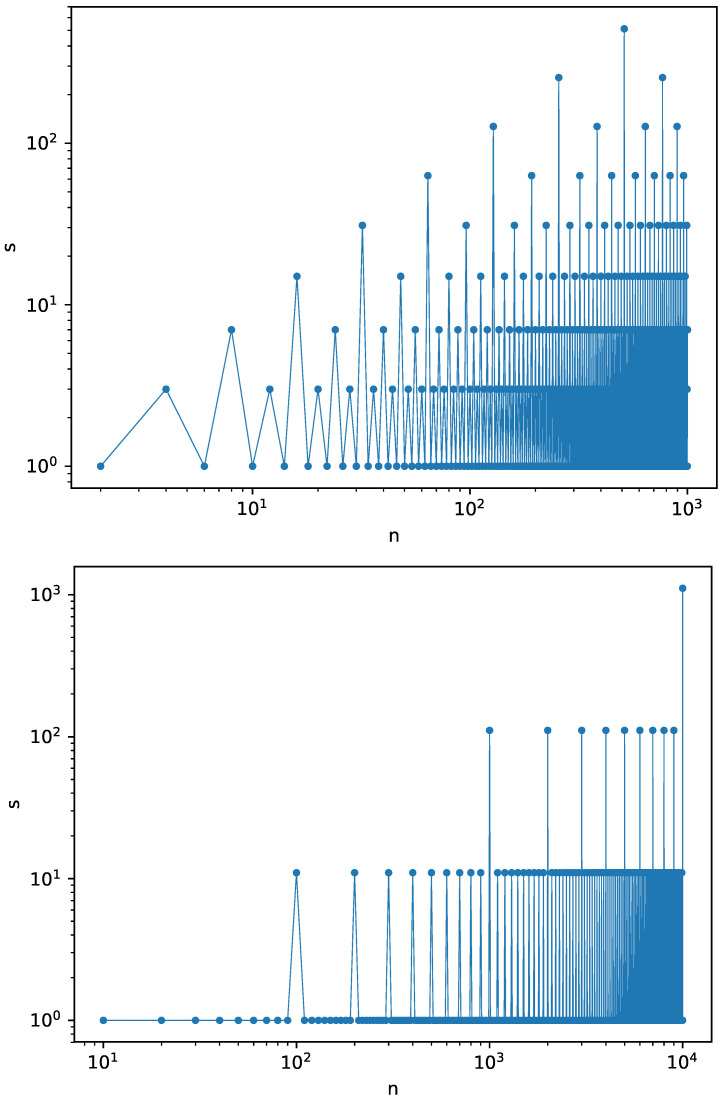
The size *s* of first avalanches for k=2 (top) and k=10 (bottom).

The plot of the dependence s(n) for b=k=2 is shown in Figure 2 (top), where *n* is the number of grains fed to the root. In Figure 2 (bottom), the sequence of a first thousand of avalanches is shown for b=k=10. The sequence for k=10 starts with 1,1,1,1,1,1,1,1,1,11,1,1,1,⋯ and it can be constructed according to the same rule as above.

It is clear from the construction that larger avalanches happen less frequently. In addition, the sizes of avalanches are allowed to be only selected numbers, namely $Q\left(w\right)=\left(1-k^{w+1}\right)/\left(1-k\right)$, where *w* is the level attained by an avalanche. The process is non-stationary. The modification of the classical approach [1] is that grains are delivered always to the same site, i.e., the root. In this sense, the process is deterministic [3]. It is more important that the size of a maximal avalanche is not limited. This assumption will be released below. Finally, the avalanches are ordered; contrary to all models of SOC we know, here we do not use any pseudorandom numbers. However, as long as only the size distribution of avalanches matters, their ordering in time does not change the results.

As a consequence of these differences, a question appears regarding how to identify the process of self-organization in our approach. We note that large avalanches cannot appear at the beginning of the process. More precisely, an avalanche of size *S* can appear for the first time only when the total number of grains attains *S*. This difficulty is weakened if we reintroduce the finite system size, for example as a maximal number of level *R*. When for some *R* we have an avalanche Q(R) for the first time, all grains are removed from the system; since then, the sequence s(n) is a periodic function of the number *n* of grains dropped at the root. In this sense, Q(R) is a characteristic scale for the number of grains, and it can be translated into the time when the grain size distribution attains its asymptotic shape. In other words, instead of ordered avalanches in a Bethe lattice, we get periodic avalanches in a Cayley tree.

**Figure 3:**
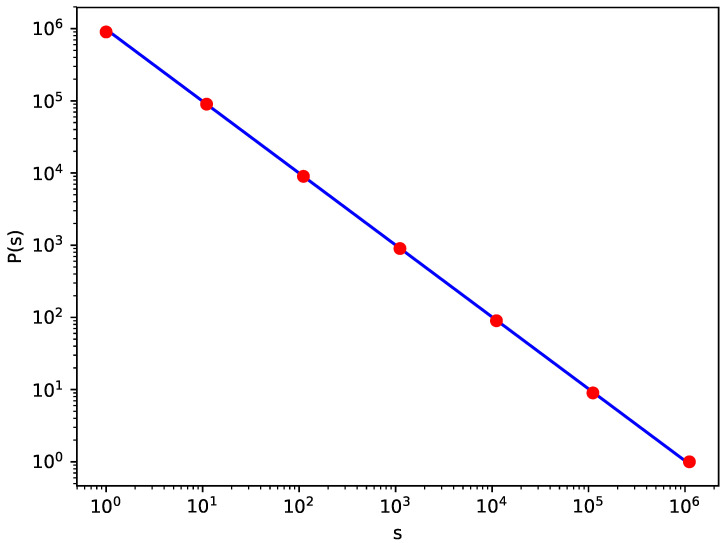
The distribution of avalanche size for k=b=10.

Coming back to the case of unlimited growth, we recall that, for s=Q(w), for the first time, n=Q(w) grains should be delivered; next, the whole sequence is repeated k−1 times with s=Q(w+1) at the end instead of Q(w). By construction, the frequency of events when s=Q(w+1) tends to Q(w)/k. In Figure 3, the avalanche size distribution is shown for k=10 and $n=10^{6}$, and the log–log plot $P\left(s\right)\propto s^{-\tau }$ is nicely linear, with the exponent τ equal to 1. On the other hand, for k>>1, the function Q(w) can be approximated as $k^{w}$, which means that Q(w+1)=kQ(w). Consequently, the mean value <s> tends to infinity with the number *n* of grains.

To demonstrate the character of these dependencies, let us consider the cutoff R>>1 again. Now, we use the probability of avalanche size *s* equal to the probability of level *w*, with P(s;R)=P(w;R) for s=Q(w), and P(s;R)=0, elsewhere. We have

(1) $$A\left(R\right)\sum_{w=0}^{R}{\left[Q\left(w\right)\right]}^{-1}=1.$$

Hence, $A\left(R\right)=1/(1-{\left(1/k\right)}^{R+1})\approx 1$. The mean size of avalanche tends to infinity linearly with *R*. For k>>1, the moments $M_{m}$ of higher order *m* are

(2) $$M_{m}\approx \sum_{w=0}^{R}{\left[Q\left(w\right)\right]}^{m-1}\approx \sum_{w=0}^{R}k^{w(m-1)}=\frac{k^{\left(m-1)(R+1\right)}-1}{k^{m-1}-1}\approx k^{R(m-1)}.$$

As the cutoff for the avalanche size is $s_{c}=\left(k^{R+1}-1\right)/\left(k-1\right)\approx k^{R}$, for m>1, the *m*th moment scales with the cutoff as $s_{c}^{m-1}$.

The obtained results on the scaling exponent of the distribution P(s) are different than in the mean field model; there, $P\left(s\right)\propto s^{-3/2}$ [13,14,15]. However, here we do not observe any indication of the upper critical dimension. As the dimension of the Bethe lattice is known to be infinite, it is higher than any critical dimension. We infer that our model should be classified as a variation of mean field theory. On a margin, we note that this model setup allows for states where two neigboring nodes are occupied by one grain each, contrary to the well-known solution for the Bethe lattice [13].

## 3. More General Approach towards an Application to Social Systems

In [16], the authors investigated sequences of online comments, and most of them were emotionally laden. Namely, the probability p(e|ne) of the next comment online as dependent on the number *n* of previous comments expressing the same kind of emotions *e* has been extracted from the BBC and Digg data. The variable *e* was introduced in [16] to classify emotional character of a comment, with e=−1,0,+1 as a negative, neutral or positive one. There, p(e|ne) for a given *e* has been found to increase with *n*, and this effect was particularly clear for e=−1. This was an indication that any psychological obstacle to give a comment decreases with the number of previous comments. In our approach, this decrease is substituted by a sharp drop of the obstacle; having obtained *b* signals, users are activated. This behaviour of users is taken directly from the sandpile models [3], where a toppling appear when the number of grains exceeds some critical value.

On the other hand, the condition b=k, adopted above, is equivalent to the conservation of the number of grains in local events. Justified in the models of sand piles, it can be too restricting in other applications, as rumor spreading [17,18] or trolling [19,20]. We are particularly interested in examples where the effect of threshold plays a role, and the signal can accumulate before it is released. The nature of the threshold can be emotional, as when an actor confronted with a new information tries to ignore it, but she gives up when she finds that the message is repeated. (For a multifaceted analysis of emotions in online communities, we refer to [21].) Then, once a message appears online, we do not expect a correlation between the number of receivers and the emotional barrier overcame by the sender.

Having this in mind, we repeat our calculation with b≠k. Namely, we assume that each node is equipped with a counter, and the signal is released when the number of grains reaches the barrier *b*. The signal is in the form of grains, and each of *k* neighbors of the sender at the next level receives one grain, no matter how large the barrier *b*.

Suppose in particular that $b=k^{2}$. For k=2, the sequence of signals is: 1,1,1,3,1,1,1,3,1,1,1,3,1,1,1,7,⋯ and so on. It is clear that the distribution of avalanches is changed because small avalanches are relatively more frequent. Actually, the condition $b=k^{2}$ means that the distribution $P\left(s\right)\propto s^{-2}$. In general, $P\left(s\right)\propto s^{-\tau }$, where

(3) $$\tau =\frac{log(b)}{log(k)}.$$

**Figure 4:**
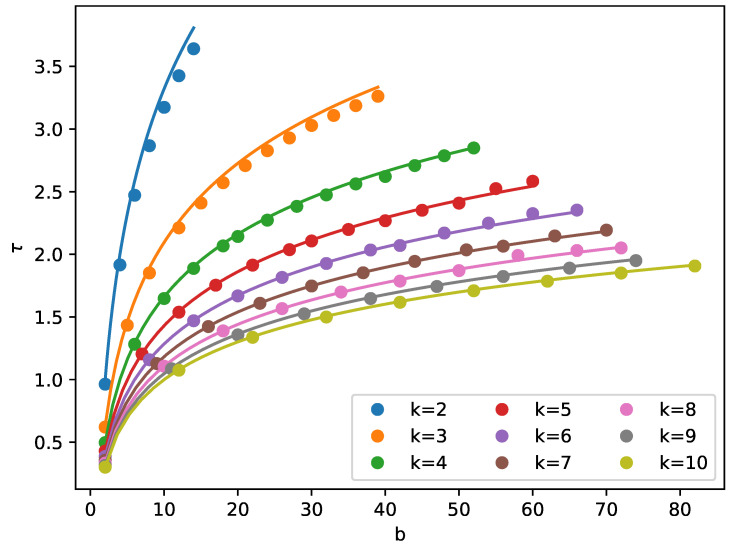
The exponent τ obtained from the simulation (points) and from Equation (3) (lines).

We note that a similar formula for the critical index has been given in [22], Equation (14), in the context of growth. In Figure 4, we have shown a comparison of this analytical formula with the simulation; the accordance is quite good. The exponent τ contributes to the scaling of the moments $M_{m}$, which—for m>τ—change with the cutoff as $s_{c}^{m-\tau }$.

In the literature, attention is also paid to the distribution of time duration of avalanches [3]. Here, this time is equivalent to the number of levels *w* reached by the avalanche. The relative frequency of a given value *w* is

(4) $$P\left(w;R\right)=P\left(Q\left(w\right);R\right)\propto {\left[Q\left(w\right)\right]}^{-\tau }={\left(\frac{k-1}{k^{w+1}-1}\right)}^{\tau },$$

and it varies with *w* approximately as $k^{-w\tau }=b^{-w}$. By construction, for given *b*, this distribution does not depend on *k*; the signal reaches subsequent shells of nodes in the same way, no matter how many nodes are in the shell. The distribution is confirmed with numerical simulations.

## 4. Fractal Properties

In order to characterize the fractal properties of the sequence of avalanches on different variants of the Bethe lattice, the multifractal analysis can be applied. In our calculations, the multifractal detrended fluctuation analysis (MFDFA) [23], which is a widely used technique for quantifying self-affinity in non-stationary time series, was applied. Within this framework, the fluctuation functions $F_{q}\left(s\right)$ are computed for multiple scales *s* and, if they follow the power-law behaviour, both the Hölder exponents α (together with the Hurst exponent as a persistence indicator) and their distributions are determined and depicted in the form of the multifractal spectrum f(α) [24]. The wider spectrum reflects a more developed multifractal and thus a more complex temporal organization of the analysed signal. For a monofractal structure, the multifractal spectrum shrinks to a single point. An important feature of the spectrum is its asymmetry quantified by an asymmetry coefficient $A_{\alpha }$ [25]. A positive value of $A_{\alpha }$ corresponds to the left-sided asymmetry of the spectrum which reflects a richer heterogeneity of scaling of large fluctuations and thus their more developed multifractality than of the smaller fluctuations. Conversely, negative $A_{\alpha }$ corresponding to the right-sided spectrum asymmetry reflects a richer heterogeneity of scaling in the domain of small fluctuations.

In the present analysis, a sequences of avalanches generated by the Bethe lattice with the number of links k∈{2,3,4,5} and the barrier b=2 is examined. Moreover, for k=2, other values of barriers i.e., 3,4, and 5 are considered as well. Within the MFDFA technique the second-order polynomial for detrending was used which ensures reliable estimation of the spectrum [26]. The *q*-values were restricted to the range [−4,4], which, in most realistic situations, allows for avoiding the divergent moments.

**Figure 5:**
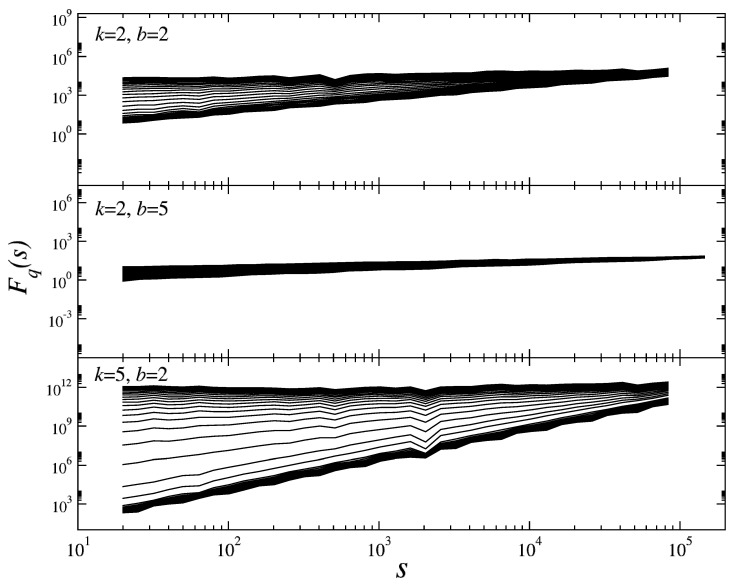
Fluctuation functions $F_{q}\left(s\right)$ estimated for the series of consecutive avalanches size for three Bethe lattices with different parameters.

In Figure 5, the fluctuation functions $F_{q}\left(s\right)$ estimated for the sequence of avalanches generated by means of the three different lattices are depicted. As is clearly seen in all the cases studied, the $F_{q}\left(s\right)$ functions reveal an almost perfect scaling spanning over four orders of magnitude in scales that provide evidence for a fractal organization of the data. However, the degree of heterogeneity of the scaling exponents depends on the lattice properties and on the barrier level. The broadest span of the scaling exponents of $F_{q}\left(s\right)$ (measured by the slopes of $F_{q}\left(s\right)$ in the log–log scale of Figure 5) is observed for the lattice with k=5 and b=2. Here, the scaling exponents at the opposite ends of the *q*-values are populated densely, however, while those that correspond to the intermediate *q*-values develop a much larger spread. This reflects multifractality, which, in addition, is strongly non-uniform [25]. In contrast, for a lattice with k=2 and b=5, the scaling approaches homogeneity, thus a monofractal organization of the corresponding series. Finally, for a process on the lattice characterized by k=b=2, the scaling of $F_{q}\left(s\right)$ is spread most evenly.

**Figure 6:**
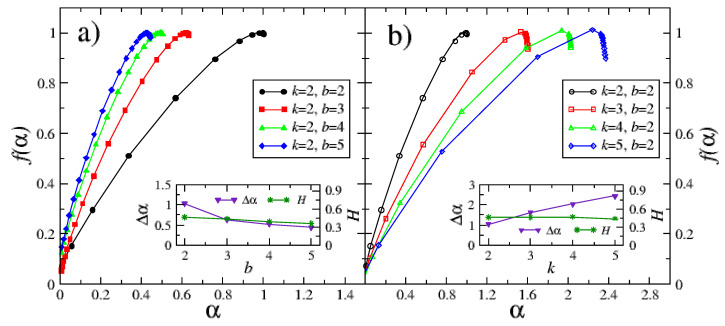
Main: multifractal spectra f(α) estimated for the sequences of avalanche sizes generated on the Bethe lattice with varying barrier *b* (**a**) and number of links *k* (**b**). Note the different scales on the *x*-axis. Insets: width of the multifractal spectrum Δα and the Hurst exponent *H* estimated from the corresponding f(α) spectra.

A more systematic sequence of the corresponding characteristics, expressed in terms of the multifractal spectra f(α), is depicted in Figure 6. All spectra are seen to develop a strong left-sided asymmetry ($A_{\alpha }\approx 1$). The impact of the number of links and of the barrier size on its specific shape is different however. The larger the barrier size *b*, the smaller the width Δα of f(α) and thus the more monofractal the organization of avalanches and also the more antipersistent is its behaviour (Figure 6a). The corresponding values of Δα and of the Hurst exponent are shown in the inset of Figure 6a as a function of *b*. The antipersistent character of the avalanche sequence is related to the fact that each avalanche larger than 1 (1 means avalanche at the first level of the lattice) is preceded and followed by the smaller one, which gives rise to anticorrelation of the entire sequence. Moreover, the larger the number of avalanches at the lattice level *n* needed to release the avalanche at the level n+1, which is determined by *b*, the stronger the antipersistence.

On the other hand, the growing number of links *k* between neighbours in the Bethe lattice contributes to widening the multifractal spectrum of avalanche series (cf. Figure 6b). However, as reflected by the *q*-dependence of the fluctuation functions $F_{q}\left(s\right)$, as discussed above, the Hölder exponents α concentrate mainly in the two extreme values indicating bi-fractality of the underlying avalanching process. The large values of α reflect a trend of the process for a fast developing cascade of large avalanches, especially for small *b*.

**Figure 7:**
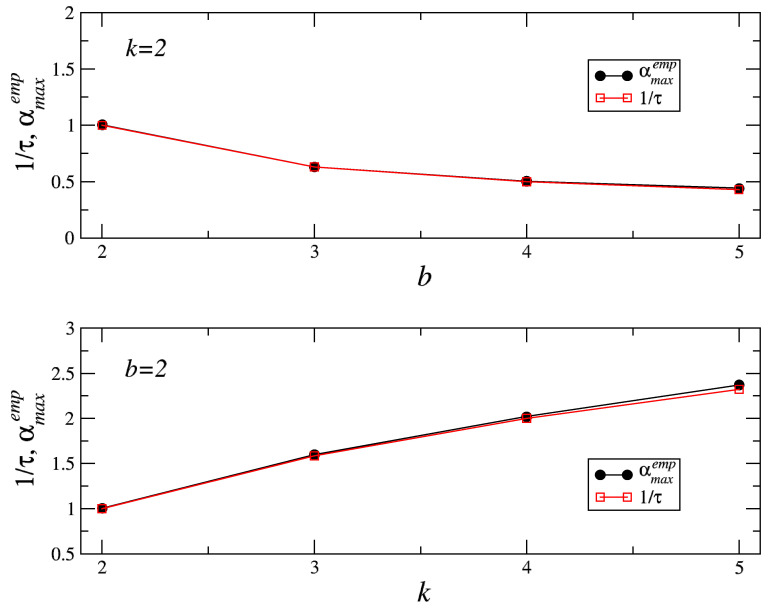
The maximal value of the scaling exponent $\alpha_{max}$ (here denoted as $\alpha_{max}^{emp}$) as read empirically from the spectra shown in Figure 6, compared with $\tau^{-1}=log\left(k\right)/log\left(b\right)$.

Based on the above results, the following formula for maximal singularity strength $\alpha_{max}$ [24] can be proposed:

(5) $$\alpha_{max}=\frac{log(k)}{log(b)},$$

where the barrier *b* can be interpreted as a time scale and *k* as singular measure. In other words, $\alpha_{max}\tau =1$. The validity of this rule is demonstrated in Figure 7.

We have verified numerically that the fluctuation functions $F_{q}\left(s\right)$ calculated from the series representing the duration of avalanches do not reveal any sufficiently convincing scaling that would indicate fractality. This in fact is consistent with the exponential distribution of avalanches’ duration time.

## 5. Discussion

The relation between the maximal singularity strength $\alpha_{max}$ and the parameter τ is similar to the one obtained for the Lévy distributions [27]. In both cases, the fractal spectrum f(α) is close to a bifractal, and the correlations are of short range. The difference (τ instead of τ+1) should perhaps be attributed to the strong discreteness of our probability distribution $P\left(s\right)\propto \delta_{s,Q(w)}$.

In some sense, our approach is close to the directed one-dimensional version of the Bak–Tang–Wiesenfeld model [1] (see also [3], p. 87). The equivalence appears because, in our system, all nodes at a given level of the Bethe lattice have the same number of grains. This equivalence is visible when we assign mass $k^{c}$ to each cell in the one-dimensional model, where *c* is the cell position, equivalent to the level in our Bethe lattice. It is clear that the avalanche distribution P(s) depends on the mass distribution, hence on the node degree. Accordingly, the topology dependence of the exponent τ is understandable. If we add the assumption on initialization localized at only one node of the lattice, the ordered character of the avalanches can also be reproduced in a one-dimensional system. In addition, the analogy is supported by the result τ=1 obtained for the conservative one-dimensional Bak–Tang–Wiesenfeld model [28].

In most models of SOC, both the avalanche size and the duration time obey the scaling relations ([3], pp. 82–83). On the contrary, here power-law is found only for the avalanche size P(s). However, the origin of these relations are different. For example, in the Bak–Sneppen model, an avalanche spreads at most linearly in time, as only two nearest neighbors of an evolving node are modified at each time step [29]. In our case, the scale-free character of the distribution of avalanche size is a consequence of the topology of the Bethe lattice. Namely, the number of newly activated nodes increases exponentially with the level number. In other words, the same scale-free character of P(s) for standard SOC and in our approach is due to two different causes.

Looking for an experimental counterpart of these results in literature, we should consider a phenomenon on a network where nodes at the same distance from the root behave in the same way: collect impulses until the same threshold and then topple. We refer again to sequence of online comments on emotionally laden threads [16,30]. There, ([30], Figure 2A) a histogram has been shown of thread length *L*, and the data have been fitted with the curve $h\left(L\right)\propto L^{-\eta }$ with η=2.5. However, the structure of the related network is rather a star with linear branches, which is far from the Bethe lattice [31]. In addition, some authors respond more than once, which makes the network structure more complex. Another example which could be relevant is the distribution of size of petitions online, where contributors can respond only once. We expect that the news about the petition spreads among a population of individuals of common interest. There, however, we cannot know who passed information about the petition to whom; such data are crucial if we want infer about the network structure. We note only some evidence for the scale-free distribution of the avalanche size: [32] (Figure 3, right, with τ≈1.0), and [33] (Figure 1, with the related exponent depending on thresholds imposed by the governmental rules; for petitions up to 10,000 signatures and for the cumulative distribution, the authors get the value 1.42). In general, a lack of information on a real structure of network makes any comparison with experiment questionable. On the other hand, our simplified model is based on the assumption of coherent avalanches, in which all topplings at nodes at the same level appear simultaneously. This is possible for man-made systems as a network of identical units, which fail when some parameter exceeds a threshold value, and the failure is transmitted to neighboring units. In particular, phenomena close to SOC systems have been discussed in systems’ electric power [34,35,36,37] and traffic [38,39,40]. Another possible application of SOC is the water management in a system of reservoirs to control floods [41,42,43]. It is worthwhile to add that our model assumption on the same threshold at each node can be released; it is enough to state that the distance to the threshold is kept at the same value at all nodes at a given level. In the case of floods, such a condition can be a consequence of minimizing the risk of a local flood. We add that the analogy of an avalanche to a spread of information, explored e.g., in [44], applies also to an epidemic with a threshold [45].

Trying to describe our model in terminology of [3], we can summarize its attributes as follows: no tuning, infinite dimension (Bethe lattice), infinitely slow driving, and order of avalanches resulting from the fully deterministic dynamics and from the network topology. As a result, we get a series of non-universal, parameter-dependent exponents related to the avalanche size distribution, an exponential probability distribution of time of duration of the avalanches, and the bifractal spectrum of the temporal signal of subsequent avalanches. Although the obtained exponent τ for the avalanche size is different than 3/2, which is commonly adopted as the result of mean-field theory, we interpret our approach as a mean field as well. Furthermore, in our approach, the Bethe lattice is akin to a one-dimensional chain because, at a given level *w*, all nodes remain in the same state. Despite this resemblance, the scale-free distribution of the avalanche size is the direct result of the topology of the Bethe lattice.

The analytical character of our results is due to the fully deterministic behaviour of avalanches and to the Bethe lattice. These assumptions can be released in various directions. In particular, topological disorder can be introduced, as in diluted Cayley trees [46], or some fraction of grains can be initially distributed randomly in the lattice [47], or new grains can be placed during the process at random nodes and not only at the root, which drives us back to the original formulation [1]. For all these approaches, our results provide a common limit case; hence, it can serve as a convenient point of reference. As long as the definition of SOC is under discussion [3], simple models like those presented here can be useful to classify some phenomena at the border of SOC. One example at the border is SOC in neural networks [48], which was claimed to be non-generic, and a term ‘self-organized quasi-criticality’ (SOqC) has been coined [49]; however, we do not claim that our model could be applicable right there.

As is known, the assumption of slow driving is not true for real systems. Basically, it can also be released here if subsequent avalanches do not interact; the next avalanche can be triggered at the root when the previous one is not finished yet. In real systems (as for example those discussed in [6]), the comments can be directly assigned to the post which initialized them, which evades the question of origin of particular comments, even if they overlap in time. If we try to generalize our approach to the case when different posts are initialized at different nodes (as it is in reality), the problem of interacting avalanches is more difficult. It is likely that additional variables should be introduced to mark the number of grains related to avalanches of given origin.

## 6. Conclusions

In conclusion, the model presented here is a new piece at the border of the mosaic of phenomena known as the Self-Organized Criticality. Instead of a stochastic series of avalanches, we have an ordered and predictable sequence of them. In a finite system, this sequence is periodic; in an infinite lattice, the size of maximal avalanche grows unbounded, but these maxima are more and more rare. The weakness of the model is that some standard features of SOC are not reproduced. Its advantage is computational simplicity, new and nontrivial results. We hope that our research strategy to bridge deterministic and stochastic phenomena will also be useful for other models of dynamics of complex systems.
